# Deletion of a single-copy DAAM1 gene in congenital heart defect: a case report

**DOI:** 10.1186/1471-2350-13-63

**Published:** 2012-08-02

**Authors:** Bihui Bao, Liang Zhang, Hua Hu, Shuxin Yin, Zhiqing Liang

**Affiliations:** 1 Department of Gynecology and Obstetrics, South-West Hospital, Third Military Medical University, Chongqing, 400038, China; 2 National Engineering Research Center for Beijing Biochip Technology, Beijing, 102206, China; 3 Department of Gynecology and Obstetrics, 261st Hospital of P.L.A, Beijing, 100094, China; 4 Department of Gynecology and Obstetrics, Chengdu Military General Hospital, Chengdu, Sichuan, 610083, China

**Keywords:** Congenital heart defect, Copy number deletion, DAAM1 gene

## Abstract

**Background:**

With an increasing incidence of congenital heart defects (CHDs) in recent years, genotype-phenotype correlation and array-based methods have contributed to the genome-wide analysis and understanding of genetic variations in the CHD population. Here, we report a copy number deletion of chromosomal 14q23.1 in a female fetus with complex congenital heart defects. This is the first description of DAAM1 gene deletion associated with congenital heart anomalies.

**Case Presentation:**

Compared with the control population, one CHD fetus showed a unique copy number deletion of 14q23.1, a region that harbored DAAM1 and KIAA0666 genes.

**Conclusions:**

Results suggest that the copy number deletion on chromosome 14q23.1 may be critical for cardiogenesis. However, the exact relationship and mechanism of how DAAM1 and KIAA0666 deletion contributes to the onset of CHD is yet to be determined.

## Background

Congenital heart defect (CHD) is the most common developmental anomaly worldwide [1]. During the first 6 or 7 weeks of pregnancy, adverse hereditary factors and an unfavorable microenvironment can both disturb fetal cardiogenesis, and such effects may even extend into the second and third trimesters [2]. Increasing evidence supports the role of genetic contributors such as chromosomal variations, submicroscopic chromosomal defects, allelic imbalance and single nucleotide mutations in individuals with CHD, especially those with additional birth defects, although the etiology remains largely unknown [1,3-5]. Therefore, testing for multiple congenital anomalies has focused on the identification of chromosomal abnormalities and basic genetic loci with critical functions in cardiac morphogenesis.

Copy-number variation (CNV) is a common submicroscopic chromosomal imbalance that generally contributes to human genetic diversity, phenotypic variation, disease or disease susceptibility [4,6] The Affymetrix® Genome-Wide Human SNP Array 6.0 have developed into a useful tool which contains more than 946,000 probes for detecting potential chromosomal defects. Using this technology, we identified a 286 kb deletion within chromosome 14q23.1 in one CHD fetus. Microdeletion on 14q23.1 contains just the DAAM1 and KIAA0666 genes, both of which are involved in cytoskeletal reorganization and morphogenetic cell movement. Moreover, the formin-homolog protein dishevelled associated activator of morphogenesis 1 (DAAM1) physically interact with Dsh, RhoA and Wnt signaling to regulate cell polarity, division and convergent extension (CE) movement, which are essential for embryonic heart development [7,8]. So we propose that a single copy-number deletion of DAAM1 may affect the spatial and temporal patterns of cardiogenesis via the Wnt signaling pathway and/or other bioeffect.

In this report, we present the clinical and molecular findings from a fetus with complex heart defects, and discuss the association of CHDs with DAAM1 deletion and prenatal environmental exposures.

## Case presentation

### Subjects and samples

Five fetuses with echocardiograms documenting CHD were recruited from the Prenatal Diagnosis Center and the Ultrasonic Diagnosis Center, Southwest hospital, Chongqing. They had a diagnosis of sporadic congenital heart defects without aneuploidies. One fetus, at 26^+2^ weeks of gestation, had dexiocardia with a single atrium, pulmonary stenosis (2.9 mm *vs.* aortic 3.5 mm in diameter), double-outlet in the right ventricle, complete endocardial cushion defect and hydropericardium (3.4 mm deep). Another fetus showed a single atrioventricular heart with transposition of the great arteries and pulmonary stenosis. The third fetus had ventricular septal defects and other anomalies in multiple internal organs. The other two fetuses had tetralogy of Fallot.

Considering the inevitable low quality of life and the negligible chance of success in cardiac surgery after birth, we obtained fetal blood specimen through cordocentesis before legally terminating the pregnancies. Heart defects were subsequently ascertained via fetal autopsy, and the myocardium was sampled concurrently for later study. After a detailed questionnaire on history of maternal disease and environmental insults during pregnancy, peripheral blood samples were collected from the parents to study patterns of CNV inheritance. For the control, venous blood was sampled from five healthy adult volunteers and eight unrelated normal neonates at random.

### Affymetrix genome-wide human SNP 6.0 analysis

Genomic DNA was isolated using the Promega Wizard DNA extraction kit (Promega Corp., Madison, WI, USA). DNA purification, digestion, ligation, fragmentation, labeling, hybridization, staining, scanning and data extraction were performed and analyzed following Affymetrix’s standard operating procedures [9].

CNVs and p value were determined from quantitative polymerase chain reaction (qPCR) probe signals (in log_2_ ratios) using the hierarchical mode analysis (HMA) algorithm [9]. DNA copy numbers less than 1.5 were regarded as deletions and more than 2.5 as duplications. All CNVs were queried in the Database of Genomic Variants ( http://projects.tcag.ca/variation/) and the UCSC Genome Browser ( http://www.genome.ucsc.edu/cgi-bin/hgGateway) [4]. Information of contained genes was queried using the Human Genome Browser (NCBI36/hg18) [9].

### Real-time quantitative PCR verification

Although 95% of presumably benign CNVs are less than 100 kb [10] in length, we validated the length of CNVs (both the ones of 20–100 kb that harbor genes known to be important for cardiac development and the ones of more than 100 kb) using a Roche Lightcycler™ automatic quantitative PCR analyzer. Primers were designed with Primer Premier 5 software and synthesized by BioSune Company (Shanghai, China). The abundance of target regions was calculated using the formula 2^(CP β actin - CP target gene)^, where CP is the crossing point in the LightCycler. Each qPCR was carried out in triplicate with the SuperGreen Fluorescence Quantitative PCR Current Kit (JingXin Technology Co., Beijing, China) and results were reported as mean ± SD.

To confirm the deletion of a single copy DAAM1 in this fetus, three pairs of primers were designed to target different regions within the deleted segment (primer set 1: LOC 58730578-58730804, primer set 2: LOC 58881650-58881950, primer set 3: LOC 58902966-58903126, NCBI36/hg18). DNA samples from the fetal blood and the myocardium were validated by technical repeats and in contrast to DNA of its parents (n = 2), the normal neonates (n = 8) and the healthy volunteers (n = 5).

## Results

**Figure 1:**
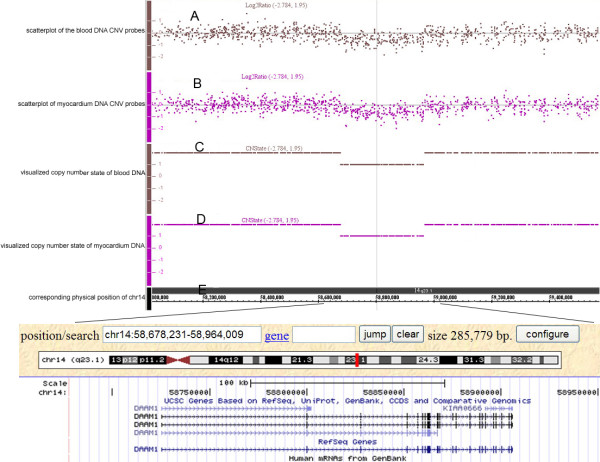
**The copy number deletion region on 14q23.1 identified by Affymetrix SNP array 6.0** ( http://www.genome.ucsc.edu/). Scatter plots with grey and purple points represented the log_2_ ratio of copy number in the fetal blood DNA (**A**) and myocardium DNA (**B**) respectively. The baseline in A and B was marked as normal copy number while the spots above the baseline means amplification and below the baseline deletion. Visualized DNA copy number in the fetal blood (**C**) and myocardium (**D**) was obtained from the mean of probe signals. Physical locus of the deletion on 14q23.1 is shown in panel E. Both DAAM1 and KIAA0666 genes were harbored (as of March 2006).

CNVs of various sizes were found in the DNA samples of the five CHD fetuses. Most of the CNVs did not contain known genes or spanned known genes without any difference from the control subjects as revealed by qPCR. A 286 kb deletion on chromosome 14q23.1 occurred in one complex CHDs with a 46,XX karyotype who had a single atrium, pulmonary stenosis, a double outlet right ventricle, dexiocardia and complete endocardial cushion defect. The physical position of this deletion spanned from 58678231 to 58964009 in the reference sequence, without overlapping any genomic variants on the BioXRT platform (version 1.03). The full lengths of the DAAM1 (LOC 58725152–58906224) and KIAA0666 (LOC 58891636–58906224) genes were mapped to the position of this CNV (Figure 1). No significant DNA variants larger than 100 kb were observed in the other four CHD fetuses.

**Figure 2:**
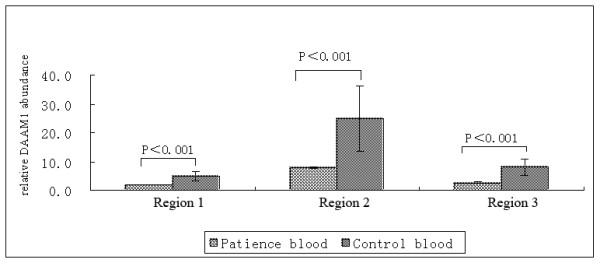
**Comparison of DAAM1 abundance between the CHD fetal blood and the control blood.** DAAM1 was significantly reduced in the three targeted genomic regions (P < 0.001). Error bars showed the standard deviation (n ≥ 3).

**Figure 3:**
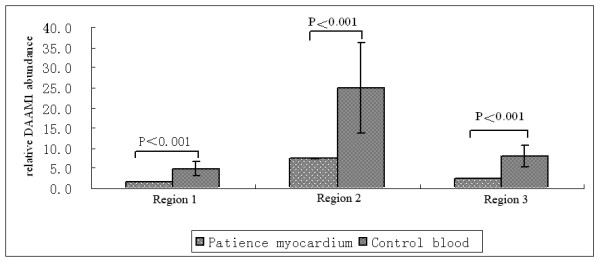
**Quantification of DAAM1 abundance in the CHD fetal myocardium and the control blood.** DAAM1 was significantly lower in the fetal myocardium than that in the control blood (P < 0.01). Error bars were the standard deviation (n  ≥ 3).

Three set of primers targeting different DAAM1 regions revealed a significant decrease of DAAM1 gene copy numbers in the CHD fetus as compared to the control subjects. Samples were then pooled together and the means and standard deviations of the relative quantity of DAAM1 gene in the control were calculated. Compared with the control, the fetal umbilical blood and myocardium DNA had a statistically significant copy number deletion in the three regions (p < 0.01, unpaired student’s *t*-test; two-tailed distribution; two-sample unequal variance Figure 2A and 2B) (Figure 3).

As for possible environmental health-risk factors, the 26-year-old parents reported that they were engaged in interior decoration where the indoor air might have been polluted. The conceived mother had also been exposed to carpets with strong chemical odors in a newly decorated office.

## Discussion

Normal cardiac morphogenesis involves the migration and shape change of primordial cells, which require exquisite regulation of gene expression and a favorable microenvironment. Genetic lesions and environmental insults such as interstitial deletion of 8p32.1 to GATA4 gene, chemicals, viral infection, or nutritional deficiency would perturb the cardiac morphogenesis and result in axial extension defects [7,11]. This study investigated whether disease-causing CNVs exist in the five CHD fetuses.

Our data did not contain any known cardiac development genes although there could be as yet unidentified genetic contributors in these CNV regions. A unique 286,000 bp deletion region harbors DAAM1 and KIAA0666 genes on chromosome 14q23.1 in one isolated complex CHDs. The genes encode scaffolding proteins. Chromosome 14q23, a genetic locus linked to the left-sided CHD, contains genes important in the vascular endothelial growth factor (VEGF) signaling pathway, such as HIF-1, PGF, ACTN1, and EIF2, which have been implicated in the cardiac valve development [4].

DAAM1 is one of the convergent-extension genes that regulates the actin cytoskeletal reorganization and plays a crucial role in CE movement of early vertebrate embryogenesis and later development [12-14]. Apical actin bundles resulted from DAAM mutation have been found much shorter and thinner than those of the wild type, and crosslinked to each other, leading to the collapse of Drosophila tracheal system and discontinuity in the tubular network [13]. Precursor cells towards heart development requires fibroblast growth factor and Wnt signaling pathway that can polarize the cytoskeleton [15]. Vertebrate Wnt signals regulates RhoA-ROK/DAAM1 to control the acto-myosin network and physically interact with both Dsh and RhoA to regulate cell polarity and cell division via the polarity complex and the formin DAAM1 respectively [16]. Via the Wnt/Fz/Dvl pathway, DAAM1 with RhoA participates in morphology and migratory behaviors in vertebrates such as cell fate specification, migration, proliferation and apoptosis [13,17]. Cardiac neural crest (cNC) passes through pharyngeal arches to the efferent pathway of the heart, and subsequent directional cell migration are essential for the formation of cardiac outflow tract [18]. Failure of the cNC directed migration may cause severe defects in the conotruncal region and the atrioventricular septum. Earlier studies found that most heart abnormalities result from delayed migration or dysdifferentiation of the neural crest cells [19]. Wnt signaling genes affect the myocardial development, and the cardiac epithelial mesenchymal transitions (EMT) relies on the Wnt/β-catenin signaling pathway [14]. Rho proteins has been found for related cellular processes, for example EMT. DAAM1 is essential for Rho activation [8]. The EMT plays a central role in the endocardial cushion morphogenesis, trabeculation and coronary system development, and it contributes to the development of mitral and tricuspid valve in the heart, as well as the maturation of interatrial and interventricular septa [7]. Based on these earlier studies, we suggest that the deletion on 14q23.1 may have a previously unidentified effect on the cardiovascular development via mechanisms relating to the primordial cardiac cells and migrating cNC cells [7]. Therefore, it is not unlikely that deletion of a single copy DAAM1, a member of the Wnt family, may be related to the causation of CHD in the fetus.

Although the role of gene dysregulation cannot be underestimated, given the complexity of heart malformations with low genetic risk in this case, the deletion of DAAM1 gene was unlikely to have produced such a profound effect. Occupational formaldehyde exposure may have contributed to the reproductive and developmental toxicity, although the potential mechanism is unclear [20]. A variety of building materials and decoration materials such as paints, coatings and adhesives release formaldehyde, benzene, radon, and ammonia, which can cause indoor air pollution and negatively affect human health especially that of the developing fetus. Gases with teratogenic effects, such as those causing of cardiogenesis disruption in developing embryos, may alter the expression of certain developmental genes involved in Wnt or VEGF signaling [4,14]. However, this is largely hypothetical and the relationship can not be determined with certainty from this study.

## Conclusions

Deletion of a single-copy DAAM1 was presumed to be causative of our CHD subject. Although we could not validated the expressional changes of DAAM1 in the fetus, we suggest that effects of DAAM1 deletion on the heart development and morphogenesis be further dissected in more CHD fetuses.

## Consent

The DNA research protocol was approved by Ethics Committee in the Third Military Medical University and the affiliated South-West Hospital in Chongqing, China. For publication of this case report, written informed consents according to protocol were granted by parents of the fetuses and newborns, as well as the healthy volunteers.

## Competing interests

The authors declare no financial or non-financial competing interests.

## Authors’ contributions

ZL sponsored the study and designed the research with LZ. HH and SY collected samples and interpreted the data. BB performed the experiments and drafted the article. LZ analyzed the data and revised the manuscript for important intellectual content. ZL approved the final version of the manuscript for publication. All authors read and approved the final manuscript.

## Pre-publication history

The pre-publication history for this paper can be accessed here:

http://www.biomedcentral.com/1471-2350/13/63/prepub
